# Solvation Free Energy Simulation for Rosmarinic Acid Extraction from *Orthosiphon stamineus*

**DOI:** 10.3390/mps2030064

**Published:** 2019-07-27

**Authors:** Cher Haan Lau, Lee Suan Chua

**Affiliations:** 1 Institute of Bioproduct Development, Universiti Teknologi Malaysia, 81310 Skudai, Malaysia; 2 Department of Bioprocess and Polymer Engineering, School of Chemical and Energy Engineering, Faculty of Engineering, Universiti Teknologi Malaysia, 81310 Skudai, Malaysia

**Keywords:** *Orthosiphon stamineus*, radical scavenging activity, solvation free energy, fragment ions, solid phase extraction

## Abstract

This study was aimed to extract rosmarinic acid from *Orthosiphon stamineus* Benth. (*Lamiaceae*) in high yield. The mixture of chloroform–ethyl acetate (70:30) was chosen as the solvent system because rosmarinic acid gave the lowest solvation free energy in that solvent system based on the computational solubility prediction. The crude extract of the plant was fractionated by C18 reversed phase absorbent to recover rosmarinic acid. The content of rosmarinic acid was increased from 4.0% *w*/*w* to 6.7% *w*/*w* after fractionation. The radical scavenging activity of rosmarinic acid rich fraction (IC_50_ = 38.3 μg/mL) was higher than the crude extract (IC_50_ = 58.85 μg/mL) based on the DPPH assay. Several phytochemicals were also identified based on the detection of fragment ions of target compounds. Fractions 1 to 3 could be combined to be a rosmarinic acid rich fraction. Simultaneously, the combination of fractions 4 to 6 could obtain a plant fraction rich in rosmarinic acid, sinensetin and eupatorin, whereas fractions 7 to 9 could be combined as a sinensetin rich fraction. The preparation of known phytochemical profile of *O. stamineus* fraction is highly required for value added product formulation and pharmacological studies, particularly for anti-diabetes and kidney related diseases which had previously been reported attributed to this herbal plant. This is the first study using solvation free energy to predict the suitable solvent system for rosmarinic acid extraction from highly complex herbal sample using the technology of solid phase extraction. The use of solvation free energy simulation is convenient and reliable before wet experiments for time and cost saving.

## 1. Introduction

*Orthosiphon stamineus* Benth. is a plant of genus *Orthosiphon* in the family *Lamiaceae*, which is widely used in traditional remedy to improve general health, treatment of kidney diseases, bladder inflammation, gout and diabetes in South East Asia countries [1]. The pharmacological effects of the plant might be due to the presence of several dominant phytochemicals such as terpenoids, polyphenols and sterols [2]. The phytochemicals of *O. stamineus* are mostly from the class of polyphenol (rosmarinic acid, caffeic acid and cichoric acid), flavonoid (sinensetin and eupatorin) and terpenoids (orthosiphol A–Z) [3].

Phytochemicals are also known as plant secondary metabolites, which are important for plant defensive system and possess important biological activity, including antioxidant activity [4]. The phenolic compounds with antioxidant property can scavenge free radicals such as hydroxyl and superoxide anion, as well as chelate metal ions [5]. The natural antioxidants from plants show the potentials in human health protection, as well as food preservatives and additives [5]. Rosmarinic acid is one of the antioxidative plant phenolics which can be abundantly found in *Lamiaceae* species [5]. *O. stamineus* was reported to have rosmarinic acid up to 53 mg/g extract [6].

Owing to the importance of rosmarinic acid in many pharmacological applications, *O. stamineus* extract was fractionated using solid phase extraction (SPE) in order to obtain a rosmarinic acid rich fraction with high antioxidant activity. The solvent system of fractionation was chosen based on the previous studies and computational solubility prediction for high yield of rosmarinic acid recovery. The chemical characteristics of this rosmarinic acid rich fraction were also evaluated in terms of the total phenolic content, total flavonoid content and antioxidant capacity compared to the crude extract of *O. stamineus.* Several target phytochemicals were also identified using high sensitivity analytical tool of LC-MS/MS. This technique is highly reliable and accurate without the use of standard chemical in compound identification.

## 2. Experimental Design

### 2.1. Plant Material

The dried whole plant of *O. stamineus* (white flower species) was supplied by Fidea Resources (Selangor, Malaysia). The stems and leaves of the plant were ground into fine powder (<1 mm) using an industrial waring blender (8010S, Biohaus Asia Sdn. Bhd, Malaysia) prior to extraction.

### 2.2. Reflux Extraction

The reflux extraction was carried out by using 70% ethanol with a solid to solvent ratio of 1:9.14 at 56.5 °C for 3 h according to the previously optimized parameters [7]. The extract was filtered and concentrated by a rotary evaporator (Heidolph, Laborota 4003, Germany) at 60 °C and further dried at 50 °C in an oven.

### 2.3. Prediction of Rosmarinic Acid Solubility by Solvation Free Energy

The solubility of rosmarinic acid was predicted using solvation free energy in order to select the appropriate solvent system for the fractionation process. Several types of mobile phases, namely, ethyl acetate, chloroform, methanol, acetic acid and formic acid were used for the pre-screening process based on the lowest solvation free energy. The solvents which were selected for the pre-screening process were sourced from previous researchers who had carried out fractionation using SPE from different types of plant species [8,9,10,11]. The calculation of solvation free energy for rosmarinic acid in different solvents was performed computationally by Forcite Package in Material Studio 7.0 (Accelrys Inc., San Diego, CA, USA).

### 2.4. Solid Phase Extraction

Solid phase extraction (SPE) was applied for the fractionation of *O. stamineus* crude extract by using a C18ec cartridge (6 mL/500 mg, Chromabond, Macherey-Nagel GmbH & Co., DUren, Germany). The pre-conditioning of SPE cartridge was performed by using methanol (6 mL), followed by equilibration with 6 mL acidified water (0.5% formic acid). One milliliter of crude extract solution (25 mg/mL) was then loaded onto the SPE cartridge. Nine fractions were collected from the SPE cartridge, where the fractions 1–8 were collected by eluting 0.2 mL of chloroform–ethyl acetate mixture in a ratio of 30:70, and fraction 9 was collected by eluting 1.0 mL of methanol for washing purpose. The collected fractions were dried at 40 °C by using an IR concentrator equipped with cold trap system (Micro-Cenvac NB 503CIR, N-BIOTEK Co. Ltd., Korea).

### 2.5. Determination of Total Phenolic and Flavonoid Content

The total phenolic content (TPC) was determined using the Folin–Ciocalteu colorimetric method according to the procedures described in the International Organization for Standardization (ISO) 14502-1 [12]. One milliliter sample (1 mg/mL) was mixed with 5 mL Folin–Ciocalteu reagent (10% *v*/*v*) and 4 mL of sodium carbonate solution (7.5% *w*/*v*). The mixture was incubated at room temperature for 60 min before the absorbance was read at 765 nm using an UV-Vis spectrophotometer ((UV-1800, Shimadzu Scientific Instruments, Japan). Blank was prepared by replacing the sample solution with distilled water. The TPC was expressed as milligram gallic acid equivalent (GAE) per 100 gdry extract.

The total flavonoid content (TFC) was determined according to the method suggested by Chua et al. [13] with some modification. One milliliter sample (1 mg/mL) was mixed with 2 mL 2% aluminum chloride methanolic solution incubated for 15 min at room temperature. The absorbance was read at 430 nm using an UV-Vis spectrophotometer. The TFC was expressed as milligram rutin equivalent (RE) per 100 g dry extract. All experiments were performed in triplicate.

### 2.6. Determination of Free Radical Scavenging Activity

The free radical scavenging activity of crude extract and its fractions from *O. stamineus* were evaluated using DPPH (2,2-diphenyl-1-picrylhydrazyl, Sigma-Aldrich, St. Louis, MO, USA) assay. The extracts were prepared at different concentrations ranging from 10 to 250 ppm. A 0.5 mL sample solution was mixed with 3.5 mL DPPH methanolic solution (0.1 mM). The samples were then allowed to stand for 30 min at room temperature in a dark place. Control was prepared by substituting the extract solution with 0.5 mL methanol. The absorbance was measured at 517 nm using an UV-Vis spectrophotometer. Ascorbic acid (≥99.5%), rosmarinic acid (≥98%)and rutin (≥94%) which were purchased from Sigma-Aldrich, USA were used as positive control. The antioxidant activity of sample was expressed as IC_50_, which is estimated at 50% inhibitory activity from the plot of inhibition against sample concentration.

(1) $$\%\;Inhibition=\left(\frac{A_{control}-A_{sample}}{A_{control}}\right)\times 100$$

### 2.7. Phytochemical Fingerprinting by Liquid Chromatography

The phytochemicals profile of the crude extract and fractions were analyzed using a liquid chromatography (Ultimate 3000) system equipped with a diode array detector (Dionex, Thermo Scientific; MA, USA) and a C18 reversed phase column (2.1 × 100 mm, 2.5 µm, XSelect High Strength Silica, Waters; Milford, MA, USA). The separation was performed using a mobile phase consisted of 0.1% formic acid in water (A) and acetonitrile (B) with a gradient elution at the flow rate of 150 µL/min: 0–10 min, 10% B; 10–25 min, 10–80% B; 25–30 min, 80% B; 30–35 min, 10% B [13]. The standard solution of rosmarinic acid was prepared in a serial concentration from 0.2 to 1.0 ppm for quantitation. Compounds were detected at 254 nm. All samples were filtered with 0.22 µm nylon filter prior to injection. Duplicate experiments were performed.

### 2.8. Targeted Phytochemicals by Liquid Chromatography Tandem Mass Spectrometry

The targeted phytochemicals were identified by an ultra-performance liquid chromatography (UPLC, Waters Acquity; Milford, MA, USA) system coupled with a triple quadrupole-linear ion trap tandem mass spectrometer (Applied Biosystems 4000 QTRAP; Life Technologies Corporation, Carlsbad, CA, USA). A C18 reversed phase Acquity column (2.1 × 150 mm, 1.7 µm) was used for compound separation. Both positive and negative modes of multiple reaction monitoring with the published transition ions were used for compound identification (Table 1).

The capillary and voltage of electrospray ionization source of mass analyzer were maintained at 400 °C and 5.5 kV (positive mode) or –4.5 kV (negative mode), respectively. The other parameters were set as follows: nitrogen was used as ion source gas for nebulization, 40 psi; for drying solvent, 40 psi; curtain gas, 10 psi; collision gas, high; collision energy, 25 to 55 eV; dwell time, 0.3 s and declustering potential, 40 V (positive mode) or –50 V (negative mode). Data acquisition and data processing were performed using Analyst 1.4.2.

The mixture of 0.1% formic acid in water (A) and acetonitrile (B) was used as mobile phases [13]. The separation was performed in a gradient elution with the following condition; 0–10 min, 10% B; 10–12 min, 10–90% B; 12–14 min, 90% B; 14–15 min, 10% B at the flow rate of 0.2 mL/min and the injection volume of 5 µL. All samples were filtered using nylon membrane (0.2 µm) prior to injection. Duplicate experiments were performed.

## 3. Results and Discussion

### 3.1. Prediction of Rosmarinic Acid Solubility by Simulation

Solubility is one of the dominant physiochemical properties to explain the performance of target phytochemical separation from extraction process. According to Savjani et al. [20], solubility is defined as the capacity of a solute to dissolve in a solvent to form a homogeneous solution. The solubility of a solute is affected by the properties of both solute and solvent, as well as environmental conditions such as pressure and temperature [20]. Thus, solubility can be applied to determine the appropriate solvent system for extraction and fractionation process. Previously, the solubility was estimated by ‘like dissolve like” rule, at which certain amount of solute is dissolved in solvent. The mutual solubility between solute and solvent should be determined by their intermolecular interactions. Ideal dissolution tends to achieve when the attraction forces of solute–solvent overcomes the attraction forces of solute–solute and solvent–solvent [21]. Hence, the solubility of a solute in a solvent can be predicted numerically from molecular structure. In this study, the magnitude of solubility between rosmarinic acid and solvents was estimated by solvation free energy calculation. The determination of solvation free energy is one of the computational approaches to estimate solubility of solute. It is widely applied in the prediction of solute aqueous solubility, as well as drug solubility. The solvation free energy is determined based on the interaction force between solvent and solute, as well as the entropy associated with creation of cavity in solvent and disruption of solvent structure. The more negative the value of solvation free energy indicates better solubility [22].

**Figure 1:**
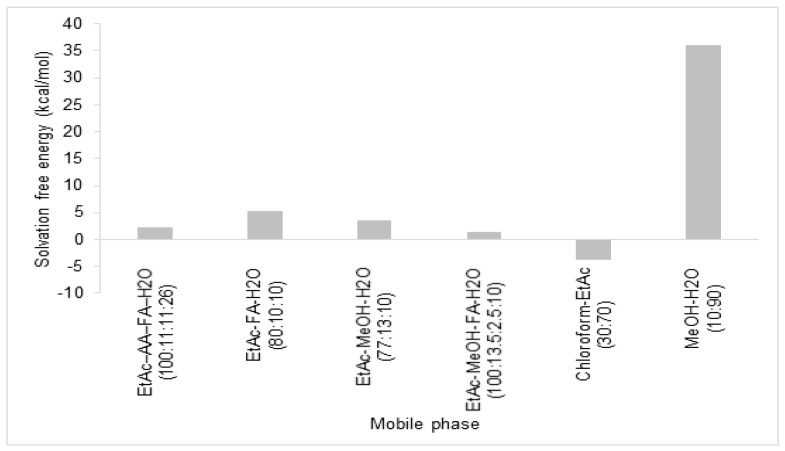
Solvation free energy of rosmarinic acid in mobile phases of fractionation.

In this study, the solvation free energy of rosmarinic acid in mobile phase for fractionation was calculated by Forcite Package in Material Studio 7.0 (Accelrys Inc., San Diego, CA, USA). The solvation free energy is the summation of the free energy of the charge removal in vacuum (ideal free energy), the free energy of addition of a neutralized molecule in the solvent (van der Waals) and the free energy of addition of charges on the solute (electrostatic). Figure 1 shows the solvation free energy of rosmarinic acid in six difference binary solvent systems. Rosmarinic acid showed to have the lowest solvation free energy in the mixture of chloroform–ethyl acetate (30:70), followed by ethyl acetate–methanol–formic acid–water (100:13.5:2.5:10), ethyl acetate–acetic acid–formic acid–water (100:11:11:26), ethyl acetate–methanol–water (77:13:10), ethyl acetate–formic acid–water (80:10:10), and methanol–water (10:90). Rosmarinic acid is an intermediate polar molecule, hence, it is more preferable in chloroform–ethyl acetate. Only this mixture of solvent system exhibited negative value of solvation free energy. Therefore, the solvent system of chloroform–ethyl acetate was selected as the mobile phase for the fractionation of rosmarinic acid due to its low solvation free energy.

### 3.2. Relationship of Rosmarinic Acid and Scavenging Activity

**Figure 2:**
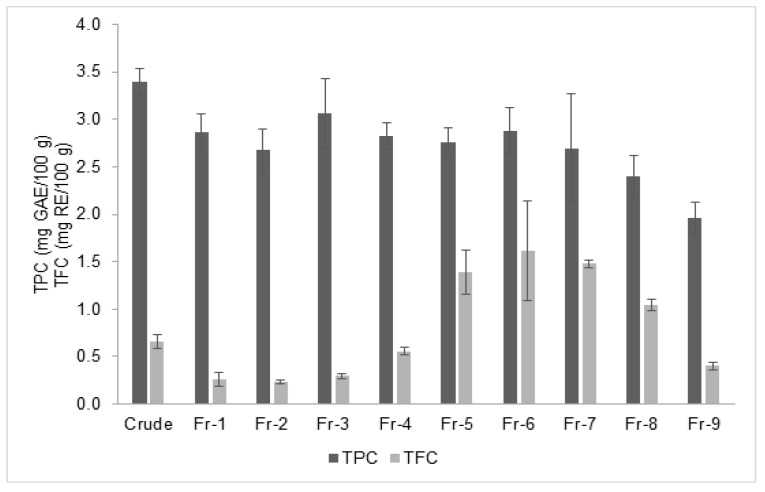
Total phenolic content (TPC) and total flavonoid content (TFC) of *Orthosiphon stamineus* crude extract and its fractions.

The total phenolic content (TPC) and total flavonoid content (TFC) of crude extract and its fractions are illustrated in Figure 2. The figure clearly shows that TPC was about 2 to 10 times higher than TFC for all fractions. The TPC of *O. stamineus* fractions varied from 1.97 to 3.06 mg GAE/100 g extract, whereas the TFC ranged from 0.23 to 1.62 mg RE/100 g extract in a bell shape curve from fractions 1 to 9.

**Figure 3:**
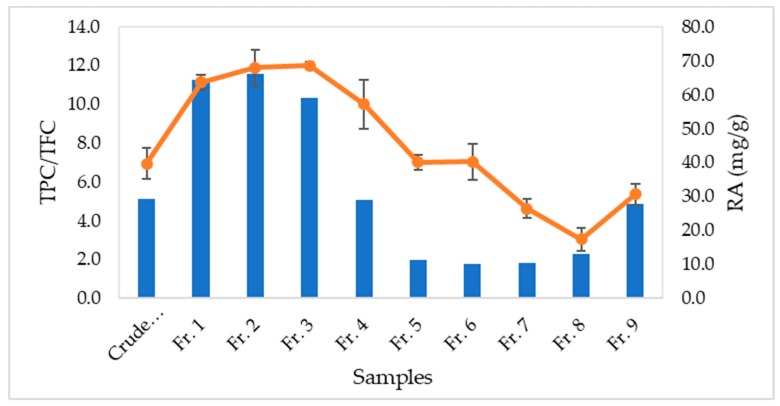
TPC/TFC ratio and rosmarinic acid (RA) content of *Orthosiphon stamineus* crude extract and fractions.

The ratio of TPC to TFC is presented in a “S” shape curve from fractions 1 to 9 in line with the concentration of rosmarinic acid (Figure 3). Rosmarinic acid seems to be the most abundant phenolic acid in *O. stamineus.* A sudden increase of rosmarinic acid content in fraction 9 was also increased the TPC/TFC ratio. This was because the remaining rosmarinic acid and other phenolic acids were rinsed out from the SPE cartridge by strong solvent, methanol.

The antioxidant activity of *O. stamineus* extract and its fractions were evaluated in term of free radicals scavenging activity and expressed in IC_50._ This IC_50_ explains the concentration of sample required to exhibit 50% of inhibition against free radicals. The phytochemicals with antiradical property could scavenge free radicals by donating protons. Rosmarinic acid could act as a potent radical scavenger, most probably because of proton donator characteristics. It has five hydroxyl groups in the molecular structure which may contribute protons to scavenge free radicals of DPPH [23].

The TPC/TFC ratio is closely correlated to the concentration of rosmarinic acid which is also highly linked to the IC_50_ of the fractions in the antioxidant assay as presented in Table 2. Hence, the TPC/TFC ratio could also be used to explain the scavenging activity of the fractions, mainly contributed by the presence of rosmarinic acid. This can be seen from lower IC_50_ values of fractions 1 to 3 with higher content of rosmarinic acid in those fractions. The increase of IC_50_ values in other fractions was also followed by the decrease of rosmarinic acid content.

In the present study, the standard chemicals of rosmarinic acid, ascorbic acid and rutin were used as positive control (Table 2). The results revealed that the scavenging capacity of rosmarinic acid and ascorbic acid were comparable because both standard chemicals showed almost similar IC_50_ values (~15 µg/mL). However, the scavenging capacity of rutin was about 3 times lower than rosmarinic acid. Therefore, rosmarinic acid was the main contributor to the high TPC/TFC ratio and high scavenging capacity of *O. stamineus* fraction in a linear relationship.

### 3.3. Major Phytochemicals in Plant Fractions

**Figure 4:**
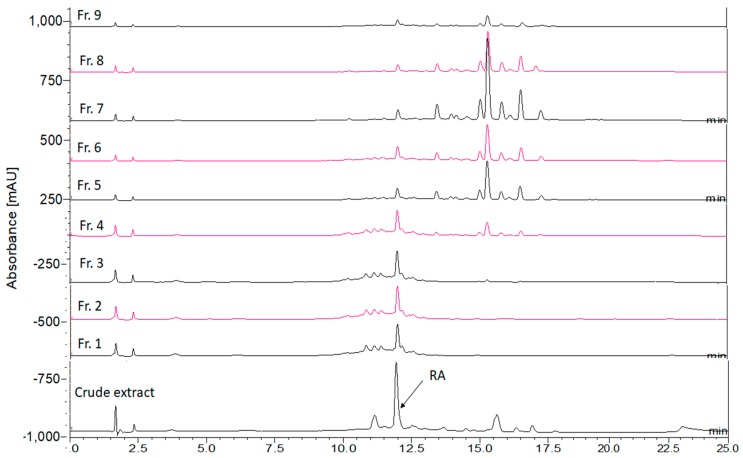
Chromatograms of *Orthosiphon stamineus;* crude extract and fractions 1–9.

Figure 4 illustrates the chromatograms of *O. stamineus* crude extract and its fractions which were obtained phytochemical fingerprinting. The fraction samples show a better HPLC separation compared to the crude extract as the undesired impurities was removed during the fractionation process. Rosmarinic acid shows the highest peak at the retention time around 12.5 min in the figure. SPE fractionation produced rosmarinic acid rich fractions 1 to 3. Further elution was found to produce rosmarinic acid mixed with other less polar compounds which could be phytochemicals from the classes of flavonoids and terpenoids. They are relatively less polar, and therefore detected at the back-end of the chromatograms. The concentration of rosmarinic acid was getting less in the subsequent fractions, whereas the other less polar compounds were increased simultaneously.

**Figure 5:**
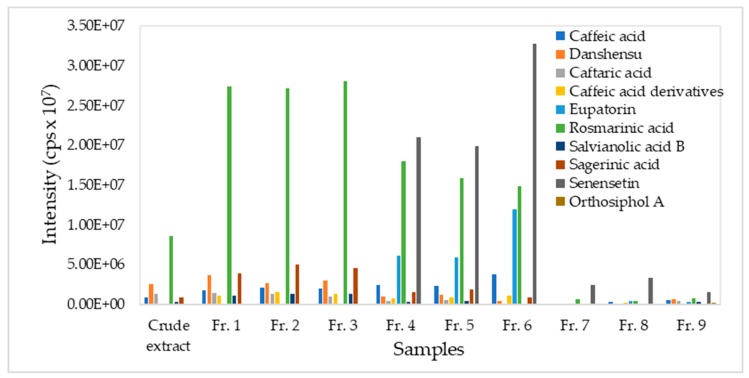
Phytochemicals detected in *Orthosiphon stamineus* fractions by UPLC-MS/MS.

Several target phytochemicals were identified in the fractions using the method of multiple reaction monitoring in liquid chromatography tandem mass spectrometer. The identification was based on the detection of characteristic ions which were previously reported in literature for *O. stamineus* extracts. The parent ions and their product ions which are listed in Table 1 were used in the target phytochemical analysis. The detected phytochemicals are presented in Figure 5 and their amount are plotted as peak area for the relative comparison, since no standard chemicals are purchased for quantitation.

Rosmarinic acid, sinensetin and eupatorin are the major phytochemicals in *O. stamineus* [24]. The presence of rosmarinic acid was detected in all fractions with decreasing concentration from fractions 1 to 9. The highest content of rosmarinic acid was detected in fractions 1 to 3. On the other hand, the intermediate fractions 4 to 6 contained significant amount of three major phytochemicals, namely sinensetin, rosmarinic acid and eupatorin in the descending order. The low rosmarinic acid in fraction 9 was not in line with the observation in Figure 3. This phenomenon could be explained by other compounds having similar retention time with rosmarinic acid in the chromatogram of fraction 9.

Other phytochemicals such as caffeic acid, danshensu, caftaric acid, caffeic acid derivative, salvianolic acid B, sagerinic acid, and orthosiphol A were also detected in lower amount in the *O. stamineus* fractions. Caffeic acid, danshensu, caftaric acid, caffeic acid derivative, salvianolic acid B and sagerinic acid showed similar trend in their concentrations along the fractions. This could be due to the high similarity of chemical characteristics of the compounds.

Based on the findings of this study, fractions 1 to 3 could be combined to obtain a rosmarinic acid rich extract (6.7%). The rosmarinic acid rich extract (fractions 1 to 3) could achieve the recovery of 57% rosmarinic acid from the crude extract. SPE fractionation also improved the rosmarinic acid content from 4.0% in the crude extract to 6.7% in the plant fraction. The fractions 4 to 6 could also be combined to obtain a plant fraction rich in rosmarinic acid, sinensetin and eupatorin. The rest of the fractions from 7 to 9 could be combined to obtain a sinensetin rich fraction.

## 4. Conclusions

The fractionation of *O. stamineus* crude extract was successfully performed using SPE to obtain plant fraction with high rosmarinic acid content. The fractionation process increased the rosmarinic acid content from 4.0% in the crude extract to 6.7% in the plant fraction using the solvent system of chloroform–ethyl acetate (70:30). The computational prediction of rosmarinic acid solubility based on the lowest solvation free energy could assist in reducing number of experimental trials for solvent selection. The binary solvent system of chloroform and ethyl acetate (30:70) increased rosmarinic acid in the plant fraction (68.63 mg/g fraction) than previously reported data (53 mg/g fraction) from this herb. The increment in rosmarinic acid content also increased the scavenging capacity of the fraction (IC_50_ = 38.29 µg/mL). Therefore, fractionation has value-added the plant extract for further investigation.

## Figures and Tables

**Table 1:** List of targeted phytochemicals and their mass fragment ions.

No.	Compounds	Molecular Weight	Mode	Fragments	Reference
1	Caffeic acid	180	Negative	179, 135	[14]
2	Danshensu	198	Negative	197, 179, 134, 123	[14]
3	Caftaric acid	312	Negative	311, 149, 179	[14]
4	Caffeic acid derivative	344	Negative	343, 161, 197, 181, 137, 135	[14]
5	Eupatorin	344	Negative	343, 328, 313, 298, 197, 161, 135	[15]
6	5-hydroxy-3’,4’,6,7-tetramethoxyflavone	358	Negative	359, 357, 358, 343, 328, 313, 299, 285, 196, 181, 162, 153	[16]
7	Rosmarinic acid	360	Negative	359, 197, 179, 161, 135	[14]
8	Salvianolic acid B (Lithospermic acid B)	718	Negative	717, 519, 339	[14]
9	Sagerinic acid	720	Negative	719, 359, 538	[14]
10	Caffeine	194	Positive	195, 163, 138, 110	[17]
11	Sinensetin	372	Positive	373, 358, 343, 185	[18]
12	Kaempferol-rutinoside	594	Positive	593, 285	[17]
13	Orthosiphol A	676	Positive	677	[19]

**Table 2:** Antioxidant activity (IC_50_) of positive controls and *Orthosiphon stamineus* fractions.

Sample	IC_50_ (μg/mL)
Rosmarinic acid	15.05 ± 2.03
Ascorbic acid	15.54 ± 1.83
Rutin	49.27 ± 6.99
Crude extract	58.85 ± 7.11
Fraction 1	37.30 ± 1.69
Fraction 2	38.29 ± 0.48
Fraction 3	39.13 ± 4.23
Fraction 4	45.10 ± 4.74
Fraction 5	71.38 ± 6.80
Fraction 6	79.53 ± 0.57
Fraction 7	82.58 ± 5.86
Fraction 8	98.56 ± 5.63
Fraction 9	74.14 ± 5.33
